# β_1_ Adrenergic Receptor Autoantibodies and IgG Subclasses: Current Status and Unsolved Issues

**DOI:** 10.3390/jcdd10090390

**Published:** 2023-09-10

**Authors:** Akane Kawai, Yuji Nagatomo, Midori Yukino-Iwashita, Ryota Nakazawa, Akira Taruoka, Yusuke Yumita, Asako Takefuji, Risako Yasuda, Takumi Toya, Yukinori Ikegami, Nobuyuki Masaki, Yasuo Ido, Takeshi Adachi

**Affiliations:** 1 Department of Cardiology, National Defense Medical College, Tokorozawa 359-8513, Japan; akanek@ndmc.ac.jp (A.K.);; 2 Department of Intensive Care, National Defense Medical College, Tokorozawa 359-8513, Japan

**Keywords:** β_1_ adrenergic receptor autoantibody, heart failure, dilated cardiomyopathy, immunoadsorption

## Abstract

A wide range of anti-myocardial autoantibodies have been reported since the 1970s. Among them, autoantibodies against the β_1_-adrenergic receptor (β_1_AR-AAb) have been the most thoroughly investigated, especially in dilated cardiomyopathy (DCM). Β_1_AR-Aabs have agonist effects inducing desensitization of β_1_AR, cardiomyocyte apoptosis, and sustained calcium influx which lead to cardiac dysfunction and arrhythmias. Β_1_AR-Aab has been reported to be detected in approximately 40% of patients with DCM, and the presence of the antibody has been associated with worse clinical outcomes. The removal of anti-myocardial autoantibodies including β_1_AR-AAb by immunoadsorption is beneficial for the improvement of cardiac function for DCM patients. However, several studies have suggested that its efficacy depended on the removal of AAbs belonging to the IgG3 subclass, not total IgG. IgG subclasses differ in the structure of the Fc region, suggesting that the mechanism of action of β_1_AR-AAb differs depending on the IgG subclasses. Our previous clinical research demonstrated that the patients with β_1_AR-AAb better responded to β-blocker therapy, but the following studies found that its response also differed among IgG subclasses. Further studies are needed to elucidate the possible pathogenic role of IgG subclasses of β1AR-AAbs in DCM, and the broad spectrum of cardiovascular diseases including HF with preserved ejection fraction.

## 1. Introduction

Heart failure (HF) is a global health and socioeconomic burden in developed countries worldwide. Dilated cardiomyopathy (DCM) is one of the most common causes of HF without an ischemic etiology and one of the most common reasons for heart transplant. In many cases, the underlying causes are unknown (hence termed “idiopathic”) [1] and may affect all ages. There has been a long debate on the pathogenetic mechanisms of DCM. According to accumulating evidence, genetic mutation, viral infection, and autoimmunity have been believed to contribute to the pathogenesis [2]. The anti-cardiac autoantibodies were discovered in the 1970s and then various kinds of autoantibodies (AAbs) such as those against myosin heavy chain [3,4,5,6,7], β_1_ adrenergic receptor (β_1_AR) [3,4,5,8,9,10,11,12], M2 muscarinic receptor [13,14,15,16,17], myosin troponin I [18,19,20,21], Na-K-ATPase and angiotensin receptor 1 (AT_1_R) have been detected to date [3,4,5]. Although the pathogenetic role of these circulating anti-cardiac AAbs has been debated, some of them have been believed to have a contributing role in the pathogenesis of cardiac dysfunction [22], Especially, among them, the autoantibody against β_1_AR (β_1_AR-AAb) is one of the most thoroughly investigated by many research groups.

The presence of β_1_AR-AAb was reported in the chronic phase of Chagas’ disease with inflammatory cardiomyopathy [23,24]. Molecular mimicry has been presumed as a mechanism of β_1_AR-AAb production since antibodies against the C-terminal of *Trypanosoma Cruzi* cross-react with β_1_AR [23,24]. The presence of β_1_AR-Aabs was reported in cardiomyopathies with specific causes, such as peripartum cardiomyopathy [25], alcoholic cardiomyopathy, and even in ischemic cardiomyopathy [26,27], and ST-elevated myocardial infraction (STEMI) with 39% positivity [28]. β_1_AR-AAbs were also detected in cardiovascular diseases other than cardiomyopathies such as idiopathic cardiac arrhythmias [29], and postural orthostatic tachycardia syndrome (POTS) [30,31].

## 2. The Basic Aspects of β_1_AR-AAb

The β_1_AR is a G-protein coupled receptor, and agonist binding produces a positive inotropic and chronotropic effect by increasing cyclic adenosine monophosphate (cAMP) in cardiomyocytes [32]. Autoantibodies directed against N-terminal, first extracellular loop, and second extracellular loop of β_1_AR were reported [33]. Among them, those against N-terminal or first extracellular loop were non-functional [9,11,33], although those against 1st extracellular loop showed minimal agonistic effect by a very sensitive detection method [34]. The β_1_AR-AAbs against the second extracellular loop of β_1_AR are regarded to have a partial agonist effect [33,35,36,37], whereas β_1_AR-AAbs showed non-competitive inhibition of radiolabeled antagonist binding to the receptor [35]. The β_1_AR-AAb showed a positive chronotropic effect on isolated rat heart myocyte in vitro [10], induced desensitization of β_1_AR [8,38,39], cardiomyocyte apoptosis [40] as well as sustained calcium influx resulting in electric instability of the heart [41,42,43]. β_1_AR-AAbs provoked by immunization of epitope peptide were also shown to induce myocardial hypertrophy and systolic dysfunction [8,44,45] accompanied by apoptosis [46] in experimental animals in vivo. The experiments using rats suggested the association between the β_1_AR-AAbs and aortic endothelial functional changes [47]. These effects were abolished by β-blocker in vitro [26,37] and in vivo [8]. The putative binding site of β-blockers differs from that of the β_1_AR-AAbs, the conformational changes of β_1_AR induced by β_1_AR-AAbs may play a significant role [35].

The possible mechanism of autoantibody production, including β_1_AR-AAbs has not been proven, although genetic predisposition to autoimmunity has been long postulated [2]. Acute heart failure was shown to be associated with B-cell activation markers [48]. A high prevalence of β_1_AR-AAb (39.1%) was reported in the patients who suffered from STEMI [28]. In the DCM patients with stage D severe HF who underwent LVAD implantation, a very high prevalence of β_1_AR-AAb (97.1%) was reported and most of them disappeared after LV unloading by LVAD support [49]. Collectively, myocardial damage resulting from pressure overload or myocardial ischemia/ infarction can lead to activation of humoral immunity and autoantibody production (See Section 4).

## 3. β_1_AR-AAbs in Human DCM

To date, anti-myocardial autoantibodies including β_1_AR-AAbs have been studied principally in patients with HF and reduced ejection fraction (HFrEF), especially in DCM. The β_1_AR-AAbs were detected in approximately 40% of DCM patients while less than 19% in healthy patients [31,50,51]. In the analysis of DCM patients with stage D severe HF who required left ventricular assist device (LVAD) implantation, as many as 34 out of 35 patients (97.1%) were positive for β_1_AR-AAb [49]. From these findings, the positivity of β_1_AR-AAb might be associated with the severity of HF. Their serum concentration was related to lower left ventricular ejection fraction (LVEF) or LV hypertrophy [26]. In clinical research conducted in the early 2000s, the presence of β_1_AR-AAbs was reported to be associated with worse clinical outcomes, such as higher incidence of cardiovascular mortality [26], fatal ventricular arrhythmias, and sudden cardiac death in patients with DCM (Table 1) [50]. These findings suggest a possible role of β_1_AR-AAbs in the pathophysiology of human DCM.

## 4. The Association of β-Blocker Therapy with β_1_AR-AAbs Mediating Pathophysiology

β-Blocker is one of the most potent agents to treat HFrEF, providing reverse remodeling of LV and drastic improvement of long-term clinical outcomes, including mortality [58,59,60]. The latest guidelines recommend prescribing β-blocker to all HFrEF patients without contraindication [61,62]. β-Blockers exert their beneficial effects by alleviating catecholamine toxicity, myocardial ischemia, oxygen consumption, and arrhythmias. Interestingly, in the randomized trials using β-blocker for HFrEF, more favorable recovery of LV systolic function and morphology in response to β-blocker therapy was observed in β_1_AR-Aab-positive patients compared to negative patients (Table 1) [53,54]. Its titer was significantly correlated with LV reverse remodeling, such as the change of LVEF, LV end-diastolic, and end-systolic volume during one-year β-blocker therapy [54]. Its titer was an independent predictor of LV reverse remodeling even after adjustment of the other confounding factors, including the target dose of β-blocker, carvedilol [54]. The other study group reported more favorable titration of β-blocker in β_1_AR-AAb-positive patients (Table 1) [52].

**Figure 1:**
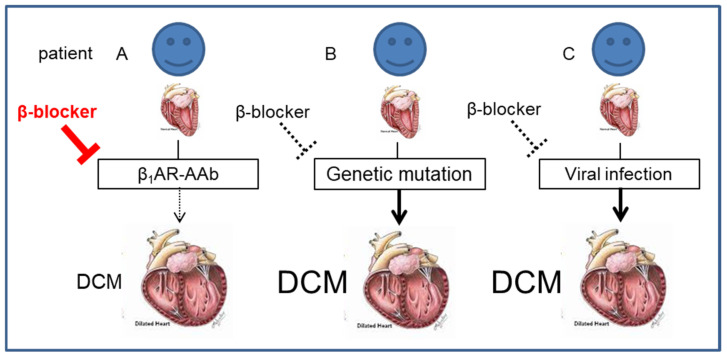
Illustration depicting the potential mechanism of more favorable reverse remodeling in β_1_ARAAb-positive patients during β-blocker therapy. β_1_AR-AAb, autoantibody against β_1_ adrenergic receptors.

The potential mechanism of more favorable reverse remodeling in β_1_AR-AAb-positive patients can be discussed as follows (Figure 1). Although genetic mutation, viral infection, and autoimmunity can play pathogenetic roles in the onset and development of DCM [2], the etiology of DCM can differ by patient. β_1_AR-AAb might play a pathogenic role in patients with β_1_AR-AAb, whereas it might be not for those who have a genetic mutation or viral infection as an etiology of DCM. Since β-blocker has been shown to be effective in inhibiting the effect of β_1_AR-AAb in basic experiments, patients with β_1_AR-AAb can benefit from β-blocker therapy. On the other hand, the effect of β-blocker may be limited since it cannot directly intervene in the genetic mutation or viral infection for those without β_1_AR-AAb (Figure 1).

**Figure 2:**
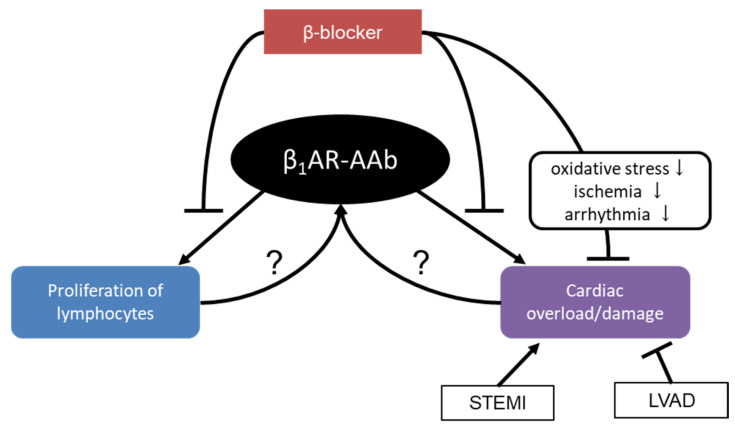
The potential mechanism of β_1_AR-AAb production and β-blockers’ effect. β_1_AR-AAb, autoantibody against β_1_ adrenergic receptors; LVAD, left ventricular assist device; STEMI, ST elevation myocardial infarction. “←” indicates promotion. “⊢” indicates inhibition.

Furthermore, the result of in vitro experiments suggested that β_1_AR-AAbs promote proliferation of lymphocytes via its agonistic effect on β_1_AR, which was inhibited by β-blocker metoprolol [63]. On the other hand, the titer and positivity of β_1_AR-AAb in HFrEF patients were reported to reduce after one year of treatment with metoprolol [52]. These results suggest that β-blocker could decrease the production of β_1_AR-AAb. However, it is not yet known whether the decrease of β_1_AR-AAbs can occur as a direct inhibition of β_1_AR-AAb production by β-blocker or as an epiphenomenon of ameliorated myocardial damage induced by β-blocker (Figure 2).

## 5. The Findings from Immunoadsorption Therapy: Proof of Concept

Immunoadsorption therapy (IA) is one of the therapeutic tools to remove anti-myocardial autoantibodies from systemic circulation through the columns. Several kinds of columns have been tested and clinical studies have shown that non-specific IA using columns loaded by sheep antihuman immunoglobulin G (IgG) or protein A improved hemodynamic data [64,65], cardiac function, and survival favorably in patients with HFrEF due to DCM (Table 1) [66,67,68]. IA might be effective in alleviating cardiac dysfunction even in congenital DCM cases [69]. Removal of β_1_AR-AAbs using IA therapy for end-stage DCM patients improved survival free from heart transplantation or LVAD [70]. Since most of these studies utilized IA columns which are not specific for β_1_AR-AAbs, it is hard to conclude the pathogenicity of β_1_AR-AAbs from these study findings. In one study, enrolling a small number of patients IA using a column specific for β_1_AR-AAbs was shown to improve LVEF and oxidative stress markers (Table 2) [71,72]. However, since there was no control group in this study, these data need to be cautiously interpreted. On the other hand, another study showed similar improvement in cardiac function (LVEF) and hemodynamics (stroke volume index and cardiac index) both in β_1_AR-AAb positive and negative groups after non-specific IA (Table 2). Thus, it is likely that not only β_1_AR-AAb but also the other kinds of AAbs (i.e., AAbs against M2 muscarinic receptor [13,14,15,16,17,73] should play a pathogenic role in DCM. A novel tryptophan column, which contains both hydrophilic and hydrophobic groups in its molecular structure and captures pathogenic substances by ionic and hydrophobic interaction, was shown to efficiently remove IgG3 subclasses [74]. A pilot study of IA using the tryptophan column showed removal of anti-myocardial AAbs including β_1_AR-AAbs and improvement of cardiac function [74], HF symptoms, and 6-min walking test distance [74]. Subsequently, Yoshikawa et al. conducted a multicenter IA study using a tryptophan column. This study randomized the study subjects into 2 groups of “IA group” which received 2-course IA treatment, and the “delayed group” which did not receive IA for 3 months and then received 1-course IA [75]. LVEF, New York Heart Association (NYHA) functional class, peak VO_2_, and 6-min walking test distance were significantly improved after IA, whereas there was no significant improvement in the non-treatment period (Table 2). Antibody score was calculated based on the enzyme-linked immunosorbent assay (ELISA) measurement of autoantibodies directed against each antigen (β_1_AR, muscarinic M2-receptor, Na-K-ATPase, troponin I, and myosin). The patients with high antibody scores showed significant improvement of LVEF and reduction of LV end-systolic volume, although those with low antibody scores did not. [75].

## 6. Controversy in β_1_AR-AAbs

There are some inconsistencies in β_1_AR-AAbs, which have been unsolved. In the basic experiment β_1_AR-AAbs from some patients increased basal and agonist-stimulated receptor activity (i.e., acted as receptor-sensitizing agents), those from other patients decreased agonist-stimulated receptor activity (i.e., acted as partial agonists) [39]. From these findings, the effect of β_1_AR-AAbs is not uniform and differs by patients. Clinically not all of the detectable β_1_AR-AAbs uniformly exert their adverse physiological effects across the spectrum of clinical conditions. While β_1_AR-AAbs were also detectable in patients with valvular or hypertensive heart disease (or even in some healthy subjects), AAbs in these non-DCM individuals were functionally inactive [81,82]. Furthermore, the presence of β_1_AR-AAb was consistently associated with increased mortality risk largely in DCM and not in ischemic cardiomyopathy [26]. For now, there have been no clues to answer the question of the inconsistencies in the physiological effect or clinical significance of β_1_AR-AAbs.

Second, it has been believed that AAbs including β_1_AR-AAbs should be produced as a consequence of autoimmunity, namely, dysregulated response to auto-antigen. However, some data suggest the possible production of β_1_AR-AAbs by cardiac damage or overload. The prevalence of β_1_AR-AAbs among the patients who suffered from the first STEMI was as high as 39.1% [28]. In the analysis of DCM patients with stage D severe HF who underwent LVAD implantation, as many as 34 out of 35 patients (97.1%) were positive for β_1_AR-AAb. More surprisingly, β_1_AR-AAb disappeared after 3 to 31 weeks following LV unloading by LVAD support in 33 of 34 patients [49] (Figure 2).

Third, β_1_AR-AAbs have been detected not only in DCM but also in a wide variety of diseases, including neuromuscular disorders [83,84] and even in periodontitis [85,86] (see Section 8), which raises the question of the specificity of β_1_AR-AAbs and this fact might imply β1AR-AAbs may serve not only as the primary causes but facilitator of a wide range of diseases.

## 7. IgG Subclasses of β_1_AR-AAbs

IgG is further classified into 4 types of IgG subclasses: IgG1, IgG2, IgG3, and IgG4, according to the constitution of Fragment crystallizable region (Fc portion). These subclasses are believed to have different functionalities in terms of their affinity to Fc receptors and potency to activate effector cells [87].

Protein A column can remove IgG1, IgG2, and IgG4 but shows low affinity to IgG3, and the anti-human IgG column can remove all IgG subclasses [78]. IA therapy with an anti-IgG column showed more improvement in LVEF, cardiac index (CI), and stroke volume index (SVi) compared to Protein A IA [78]. Furthermore, protein A IA, with the application of an improved treatment regimen for IgG3 removal, was superior to conventional protein A in terms of improvement of CI and LVEF after IA (Table 1) [79]. Some studies using the tryptophan column, which efficiently removes the IgG3 subclass, reported that the titer of IgG3 anti-myocardial AAbs, including β_1_AR-AAbs, was correlated with improvement of cardiac function after IA (Table 1). However, the titer of total IgG AAbs did not show such a correlation. [80] DCM patients with high titer of IgG3 anti-myocardial AAbs responded more favorably to IA therapy [75]. These results suggest that the IgG3 subclass might be more relevant to the pathology of DCM, and the pathogenetic roles of anti-myocardial AAbs, including β_1_AR-AAbs might differ depending on their IgG subclasses.

**Figure 3:**
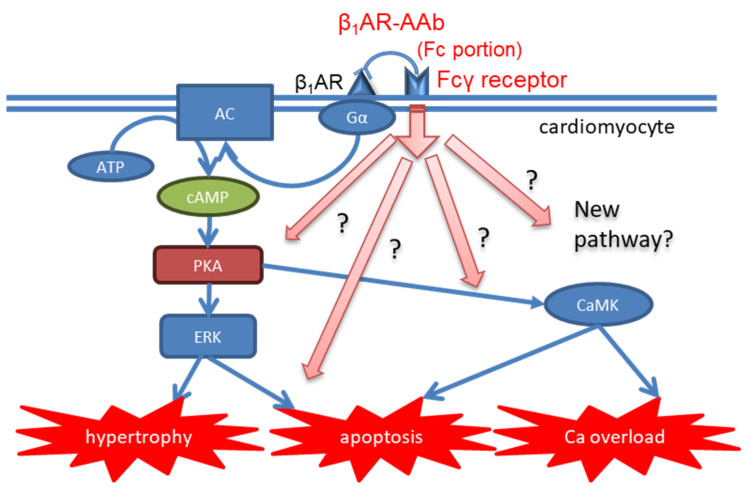
Proposed intracellular pathway interfering β_1_AR signal transduction by Fc portion binding to Fc receptor. β_1_AR-AAb, autoantibody against β_1_ adrenergic receptor; AC, adenylate cyclase; ATP, adenosine triphosphate, cAMP, cyclic adenosine monophosphate; PKA protein kinase A; ERK, extracellular signal-regulated kinase; CaMK, calmodulin kinase.

According to the previous publication, the F(ab’) fragment of β_1_AR-AAbs did not show an agonistic effect [42]. The same phenomenon was reported in AAbs against β_2_AR as well [88]. Further, Staudt et al. nicely demonstrated the essential role of Fc portion in certain kinds of anti-myocardial AAbs by in vitro experiment. Whereas the F(ab’)_2_ portion did not solely show any physiological effect of AAb, the addition of the anti-F(ab’) antibody to the F(ab’)_2_ portion successfully reproduced its physiological effect [89]. Interestingly, Fcγ receptor IIa polymorphism, which affects the binding of IgG Fc portion to the receptor, was associated with different efficacy of IA in terms of the improvement of LVEF [90]. From this finding, the interaction of the IgG Fc portion to the Fcγ receptor might play a significant role in the pathology of DCM. For example, it is possible the interaction between the Fc portion and the Fc receptor might interfere with the downstream signaling of β_1_AR, but this hypothesis has not yet been investigated (Figure 3).

The clinical study using β-blockers in the contemporary HF population also suggests the different significance of β_1_AR-AAbs by IgG subclasses. In the single-center HFrEF cohort with 96% β-blocker administration at baseline, the presence of IgG3-β_1_AR-AAb was associated with more favorable outcomes defined as the composite endpoint of all-cause mortality, cardiac transplantation, or HF hospitalization, whereas total IgG-β_1_AR-AAb failed to discriminate long-term outcomes (Table 1) [27]. The IMAC (Intervention in Myocarditis and Acute Cardiomyopathy) -2 study was a multicenter trial that was originally conducted to explore the determinants of LV reverse remodeling and clinical outcomes in patients with recent-onset cardiomyopathy. In this study, which enrolled 373 patients with recent-onset cardiomyopathy, IgG3-β_1_AR-AAb was associated with more favorable myocardial recovery during 6-month guideline-directed medical therapy, including β-blockers (94% at 6 months) [57]. The titer of IgG3-β_1_AR-AAb showed a significant positive correlation with LVEF at 6 months, whereas total IgG did not show such correlation. The presence of IgG3-β_1_AR-AAb was an independent predictor of LVEF at 6 months, as well as for 6-month change in LVEF, even after adjusting for covariates that were used in the IMAC-2 main study (Table 1) [57].

## 8. β_1_AR-AAbs in the Other Cardiac Diseases

As described above, the significance of β_1_AR-AAbs has been investigated principally in DCM patients. On the other hand, recently several studies reported the presence of β_1_AR-AAbs in patients with other cardiac diseases.

Several studies reported that β_1_-ARAAbs were detected with a certain probability in ischemic cardiomyopathy, although its positivity was reported to be lower compared to DCM [12,26,27]. The prognostic significance of β_1_AR-AAb in ischemic cardiomyopathy might be questionable, whereas it was consistently associated with increased mortality risk in DCM [26]. The prevalence of β_1_AR-AAbs among the patients who suffered from the first STEMI was 39.1%, and the positive β_1_-ARAAbs was associated with the remodeling of the left ventricle and prediction of major adverse cardiac events [28]. In the acute coronary syndrome (ACS) cohort, STEMI was associated with a higher titer of β_1_AR-AAbs [91]. However, the prognostic significance of β_1_AR-AAbs in ACS was obscure [91]. As mentioned above (see Section 6), β_1_AR-AAbs were also detectable in patients with valvular or hypertensive heart disease, but AAbs in patients with these non-DCM individuals were functionally inactive [81,82].

Some studies reported the association of anti-cardiac autoantibodies with ventricular or supraventricular tachyarrhythmias, including atrial fibrillation (AF) [29] and inappropriate sinus tachycardia [92]. The serum level of β_1_AR-AAbs among nonvalvular AF patients or lone paroxysmal AF patients was significantly higher than healthy controls [93,94]. Further, the serum level of β_1_AR-AAbs was higher in persistent AF compared with paroxysmal AF [93]. AF is the common comorbidity in hyperthyroidism. The frequency of β_1_AR-AAbs was extremely higher in patients with AF (94%) compared with sinus rhythm (38%) among the patients with hyperthyroidism such as Grave’s disease [95]. On the other hand, patients with toxic multinodular goiter or subacute thyroiditis had a low prevalence of β_1_AR-AAbs (approximately 20%) [96]. Induction of β1AR-AAbs aggravated atrial electrical instability and atrial fibrosis in rabbits. Further, in vitro experiments revealed that β_1_AR-AAb induced calmodulin kinase and ryanodine receptor 2 activation in atrial cardiomyocytes and the myofibroblasts phenotype formation [97]. β_1_AR-AAbs with thyroid hormone supplementation induced sustained AF in rabbits. AF induction was blocked acutely by the neutralization of these antibodies with immunogenic peptides despite continued hyperthyroidism. The atrial effective refractory period measured by the electrophysiological study as one parameter of AF propensity shortened significantly after immunization and was acutely reversed by peptide neutralization [98].

POTS is caused by inappropriate elevation of heart rate when patients are standing up from a prone position. While POTS patients present with a wide variety of symptoms, such as dizziness and palpitation, its detailed mechanisms are still unknown. Induction of β_1_AR-AAbs in rabbits resulted in a greater increase in heart rate during the tilt test compared with preimmune baseline, which indicated the association of β_1_AR-AAbs with POTS [99]. The G-protein coupled autoantibodies against 4 subtypes of adrenergic receptors and 5 subtypes of muscarinic acetylcholine receptors were detected in the serum of the POTS patients [30,100].

It has been reported that β_1_AR-AAbs were detected in patients with neuromuscular disorders such as chronic fatigue syndrome [84], and myasthenia gravis [83]. However, it is still uncertain whether β_1_AR-AAbs have a causal relationship with these diseases. β_1_AR-AAbs were detected even in periodontitis patients [85,86], and β_1_AR-AAbs extracted from periodontitis patients were functionally active and showed a apoptotic effect on rat atrial cells, which suggests a potential link between periodontitis and cardiovascular diseases, including HF [86].

## 9. Future Perspective

As mentioned above, the constitution of the Fc portion differs by each IgG subclass, which might lead to different interactions of the Fc portion with Fc receptors. These might contribute to their different effects among IgG subclasses. This can be a potential mechanism (Figure 3), but there have been no data supporting this hypothesis, which needs to be tested by further examinations.

In a recent study, a vaccine targeting β_1_AR is reported to be effective for reducing systolic blood pressure, attenuating myocardial hypertrophy, improving cardiac function, and reducing cardiac fibrosis and inflammation in animal disease models [101]. Studies are currently ongoing to determine if an aptamer for neutralizing β_1_AR-AAbs may curtail disease progression and perhaps even facilitate recovery [102,103].

There has been a paucity of data supporting the pathogenic role of β_1_AR-AAbs in cardiovascular diseases other than DCM. Currently, the incidence and prevalence of HF with preserved EF (HFpEF) show a drastic increase in developed countries mainly due to advanced aging society, which constitutes a predominant cause of the recent HF pandemic [104]. The presence and significance of β_1_AR-AAbs have not yet been explored in the HFpEF population. Thus, further research is needed for a better understanding of the role of β_1_AR-AAbs in the broad spectrum of cardiovascular diseases, including HFpEF.

## 10. Translational Outlook

The novel therapeutics eliminating or neutralizing the effect of β_1_AR-AAbs can be a promising approach to the treatment of HF due to DCM. For this approach, β-blocker, IA, and aptamer could be applied. For targeting the IgG3 subclass, inhibition of Fc portion—Fc receptor interaction might be a promising approach. Immunoglobulin or Fc fragment could be applied for this purpose, although its efficacy is completely unknown at present. Since a variety of anti-cardiac AAbs are detected and β_1_AR-AAbs might not be the only one that facilitates disease progression, the approach suppressing B cell and autoantibody production as a whole might be efficacious. For this approach anti-CD20 monoclonal antibody (rituximab) [105] might be promising [106].

## 11. Conclusions

The β_1_AR-AAbs have been investigated mainly in DCM patients. However, there has been a paucity of data on the disparity of β_1_AR-AAbs by IgG subclasses and the significance of β_1_AR-AAbs in other cardiac diseases, especially HFpEF. Further studies are needed to elucidate the possible pathogenic role of IgG subclasses of β_1_AR-AAbs in DCM and the broad spectrum of cardiovascular diseases, including HFpEF.

## Figures and Tables

**Table 1:** Summary of the clinical studies exploring the association of β_1_AR-AAb with clinical outcome in patients with heart failure.

Study	Study Design	No. of Patients	% β-B	Outcome in β_1_AR-AAb Positive Group (vs. Negative Unless Otherwise Specified)		The Other Findings
				IgG3	Non-IgG3	
Iwata et al. [50]	Obs	104	27%	VT↑ at baseline, cardiac death↑, sudden cardiac death↑ during 31 M		
Miao et al. [52]	Obs	96	100%	Better uptitration of metoprolol LVEF↑, LVEDD↓, LVESD↓ after 1 year		β_1_AR-AAb frequency↘ after 1 year
Nagatomo et al. [53]	Int	82	100%	LVEF↑, LVEDD↓, LVESD↓ after 16 weeks		
Nagatomo et al. (J-CHF study) [54]	Int	117	100%	LVEF↑, LVEDV↓, LVESV↓ after 14 M		β_1_AR-AAb titer correlated with ΔLVEF, ΔLVEDV, ΔLVESV
Stork et al. [26]	Obs	DCM: 65 ICM: 40	DCM: 26% ICM: 13%	All-cause death↑, cardiovascular death↑ in DCM but not in ICM		
Pei et al. [55]	Obs	DCM: 704 ICM: 1054	DCM: 77% ICM: 70%	All-cause death↑, sudden cardiac death↑ both in DCM and ICM		
Mavrogenis et al. [56]	Obs	2256	83.4%	HF rehospitalization↑		
Nagatomo et al. (NORDIC-ARCTIC study) [27]	Obs	116	97%	Better event free survival from the primary endpoint ^1^ compared to non-IgG3	Worse event free survival from the primary endpoint ^1^ compared to IgG3	
Nagatomo et al. (IMAC-2 study) [57]	Obs	373	94%	LVEF↑ compared to negative and non-IgG3 Better evet free survival from the primary endpoint ^1^ compared to negative in NYHA III-IV subgroup	LVEF↓, LVEDD↑, LVESD↑ compared to negative and IgG3 Worse evet free survival from the primary endpoint ^1^ compared to IgG3 in NYHA III-IV subgroup	IgG3-β_1_AR-AAb titer correlated with LVEF at 6 M IgG3-β_1_AR-AAb independently associated with LVEF at 6 M and ΔLVEF

^1^ the primary endpoint was defined as the composite of all-cause death, cardiac transplantation, or hospitalization due to exacerbation of heart failure. β_1_AR-AAb, autoantibody against β_1_-adrenergic receptor; %β-B, percentage of β-blocker administration; IgG, immunoglobulin G; Obs, observational study; Int, interventional study; ↓, lower; ↑, higher; ↘, decrase; VT, ventricular tachycardia; M, month; LVEF, left ventricular ejection fraction; LVEDD, left ventricular end-diastolic dimension; LVESD, left ventricular end-systolic dimension; LVEDV, left ventricular end-diastolic volume; LVESV left ventricular end-systolic volume; DCM, dilated cardiomyopathy; ICM, ischemic cardiomyopathy; Δ, absolute change; NYHA, New York Heart Association functional classes.

**Table 2:** Immunoadsorption studies suggesting the importance of anti-cardiac AAbs, β_1_AR-AAbs and IgG3 subclass.

Study	Study Design	IA Column	No. of Patients	Duration of Follow Up	Findings
Muller J et al. [66]	RCT	Anti-human IgG	34 (control 17)	12M	β_1_AR-AAb reduction, LVEF↑, NYHA↓ after IA
Felix SB et al. [65]	RCT	Anti-human IgG	18 (control 9)	3M	β_1_AR-AAb reduction, LVEF↑, CI↑, SVi↑, SVR↓, NYHA↓
Wallukat G et al. [71]	No control	Peptide column specific for β_1_AR-AAbs	8	12M	LVEF↑, LVEDd↓
Cooper LT et al. [76]	No control	Protein A	4	6M	LHFQ↓, SF36↑
Mobini R et al. [77]	No control β_1_AR-AAb (+) vs. (-) groups	Anti-human IgG	22	3M	LVEF↑, SVi↑, CI↑ both in β_1_AR-AAb (+) and (-) groups
Staudt A et al. [78]	Anti-human IgG vs. protein A column	Anti-human IgG (high affinity to IgG3) vs. Protein A (low affinity)	18	3M	LVEF↑, CI↑, SVi↑ and SVR↓ in anti-human IgG group
Staudt A et al. [79]	Protein A vs. protein A + improved IgG3 removal	Protein A	18	3M	LVEF↑, CI↑, SVi↑ and NYHA↓ in improved IgG3 elimination group
Nagatomo Y et al. [74]	No control	Tryptophan (IgG3 specific)	16	3M	β_1_AR-AAb, IgG3 reduction, LVEF↑, BNP↓, distance of 6MWT↑
Baba A et al. [80]	No control	Tryptophan (IgG3 specific)	16	3M	Pre IA titer of IgG3 AAbs correlated with the change of LVEF
Yoshikawa T et al. [75]	2 course IA vs. delayed 1 course IA	Tryptophan (IgG3 specific)	33	12M	LVEF↑, BNP↓, peak VO_2_↑ distance of 6MWT↑ after IA

IA, Immunoadsorption; AAb, autoantibody; β_1_AR, β_1_ adrenergic receptor; RCT, randomized control study; M, months; LVEF, left ventricular ejection fraction; LVEDD, left ventricular end-diastolic dimension; CI, Cardiac Index; SVi, stroke volume index; SVR, systemic vascular resistance; NYHA, New York Heart Association functional classes; ↓, decrease; ↑, increase; LHFQ, Minnesota Living with Heart Failure questionnaire; SF-36, Medical Outcomes Study Health Status Survey questionnaire; 6MWT, 6 min walking test; AAb autoantibody.

## Data Availability

The study did not report any data.

## References

[1] Maron B.J., Towbin J.A., Thiene G., Antzelevitch C., Corrado D., Arnett D., Moss A.J., Seidman C.E., Young J.B. (2006). Contemporary definitions and classification of the cardiomyopathies: An American Heart Association Scientific Statement from the Council on Clinical Cardiology, Heart Failure and Transplantation Committee; Quality of Care and Outcomes Research and Functional Genomics and Translational Biology Interdisciplinary Working Groups; and Council on Epidemiology and Prevention. Circulation.

[2] Mestroni L., Krajinovic M., Severini G.M., Pinamonti B., Di Lenarda A., Giacca M., Falaschi A., Camerini F. (1994). Familial dilated cardiomyopathy. Br. Heart J..

[3] Caforio A.L., Grazzini M., Mann J.M., Keeling P.J., Bottazzo G.F., McKenna W.J., Schiaffino S. (1992). Identification of alpha- and beta-cardiac myosin heavy chain isoforms as major autoantigens in dilated cardiomyopathy. Circulation.

[4] Schultheiss H.P., Schulze K., Schauer R., Witzenbichler B., Strauer B.E. (1995). Antibody-mediated imbalance of myocardial energy metabolism. A causal factor of cardiac failure?. Circ. Res..

[5] Limas C.J., Goldenberg I.F., Limas C. (1989). Autoantibodies against beta-adrenoceptors in human idiopathic dilated cardiomyopathy. Circ. Res..

[6] Caforio A.L., Goldman J.H., Baig M.K., Haven A.J., Dalla Libera L., Keeling P.J., McKenna W.J. (1997). Cardiac autoantibodies in dilated cardiomyopathy become undetectable with disease progression. Heart.

[7] Goldman J.H., Keeling P.J., Warraich R.S., Baig M.K., Redwood S.R., Dalla Libera L., Sanderson J.E., Caforio A.L., McKenna W.J. (1995). Autoimmunity to alpha myosin in a subset of patients with idiopathic dilated cardiomyopathy. Br. Heart J..

[8] Iwata M., Yoshikawa T., Baba A., Anzai T., Nakamura I., Wainai Y., Takahashi T., Ogawa S. (2001). Autoimmunity against the second extracellular loop of beta(1)-adrenergic receptors induces beta-adrenergic receptor desensitization and myocardial hypertrophy in vivo. Circ. Res..

[9] Magnusson Y., Marullo S., Hoyer S., Waagstein F., Andersson B., Vahlne A., Guillet J.G., Strosberg A.D., Hjalmarson A., Hoebeke J. (1990). Mapping of a functional autoimmune epitope on the beta 1-adrenergic receptor in patients with idiopathic dilated cardiomyopathy. J. Clin. Investig..

[10] Magnusson Y., Wallukat G., Waagstein F., Hjalmarson A., Hoebeke J. (1994). Autoimmunity in idiopathic dilated cardiomyopathy. Characterization of antibodies against the beta 1-adrenoceptor with positive chronotropic effect. Circulation.

[11] Magnusson Y., Hjalmarson A., Hoebeke J. (1996). Beta 1-adrenoceptor autoimmunity in cardiomyopathy. Int. J. Cardiol..

[12] Jahns R., Boivin V., Siegmund C., Inselmann G., Lohse M.J., Boege F. (1999). Autoantibodies activating human beta1-adrenergic receptors are associated with reduced cardiac function in chronic heart failure. Circulation.

[13] Sterin-Borda L., Gorelik G., Borda E.S. (1991). Chagasic IgG binding with cardiac muscarinic cholinergic receptors modifies cholinergic-mediated cellular transmembrane signals. Clin. Immunol. Immunopathol..

[14] Fu L.X., Magnusson Y., Bergh C.H., Liljeqvist J.A., Waagstein F., Hjalmarson A., Hoebeke J. (1993). Localization of a functional autoimmune epitope on the muscarinic acetylcholine receptor-2 in patients with idiopathic dilated cardiomyopathy. J. Clin. Investig..

[15] Wallukat G., Fu H.M., Matsui S., Hjalmarson A., Fu M.L. (1999). Autoantibodies against M2 muscarinic receptors in patients with cardiomyopathy display non-desensitized agonist-like effects. Life Sci..

[16] Baba A., Yoshikawa T., Fukuda Y., Sugiyama T., Shimada M., Akaishi M., Tsuchimoto K., Ogawa S., Fu M. (2004). Autoantibodies against M2-muscarinic acetylcholine receptors: New upstream targets in atrial fibrillation in patients with dilated cardiomyopathy. Eur. Heart J..

[17] Del Corsso C., de Carvalho A.C., Martino H.F., Varanda W.A. (2004). Sera from patients with idiopathic dilated cardiomyopathy decrease ICa in cardiomyocytes isolated from rabbits. Am. J. Physiol. Heart Circ. Physiol..

[18] Shmilovich H., Danon A., Binah O., Roth A., Chen G., Wexler D., Keren G., George J. (2007). Autoantibodies to cardiac troponin I in patients with idiopathic dilated and ischemic cardiomyopathy. Int. J. Cardiol..

[19] Doesch A.O., Mueller S., Nelles M., Konstandin M., Celik S., Frankenstein L., Goeser S., Kaya Z., Koch A., Zugck C. (2011). Impact of troponin I-autoantibodies in chronic dilated and ischemic cardiomyopathy. Basic Res. Cardiol..

[20] Halley C.M., Lappe J.M., Cotleur A.C., Shrestha K., Borowski A.G., Pelfrey C., Ransohoff R., Tang W.H. (2011). Antiinflammatory autoimmune cellular responses to cardiac troponin I in idiopathic dilated cardiomyopathy. J. Card. Fail..

[21] Miettinen K.H., Eriksson S., Magga J., Tuomainen P., Kuusisto J., Vanninen E.J., Turpeinen A., Punnonen K.R., Pettersson K., Peuhkurinen K.J. (2008). Clinical significance of troponin I efflux and troponin autoantibodies in patients with dilated cardiomyopathy. J. Card. Fail..

[22] Nagatomo Y., Tang W.H. (2014). Autoantibodies and cardiovascular dysfunction: Cause or consequence?. Curr. Heart Fail. Rep..

[23] Ferrari I., Levin M.J., Wallukat G., Elies R., Lebesgue D., Chiale P., Elizari M., Rosenbaum M., Hoebeke J. (1995). Molecular mimicry between the immunodominant ribosomal protein P0 of Trypanosoma cruzi and a functional epitope on the human beta 1-adrenergic receptor. J. Exp. Med..

[24] Smulski C., Labovsky V., Levy G., Hontebeyrie M., Hoebeke J., Levin M.J. (2006). Structural basis of the cross-reaction between an antibody to the Trypanosoma cruzi ribosomal P2beta protein and the human beta1 adrenergic receptor. FASEB J. Off. Publ. Fed. Am. Soc. Exp. Biol..

[25] Liu J., Wang Y., Chen M., Zhao W., Wang X., Wang H., Zhang Z., Zhang J., Xu L., Chen J. (2014). The correlation between peripartum cardiomyopathy and autoantibodies against cardiovascular receptors. PLoS ONE.

[26] Stork S., Boivin V., Horf R., Hein L., Lohse M.J., Angermann C.E., Jahns R. (2006). Stimulating autoantibodies directed against the cardiac beta1-adrenergic receptor predict increased mortality in idiopathic cardiomyopathy. Am. Heart J..

[27] Nagatomo Y., Li D., Kirsop J., Borowski A., Thakur A., Tang W.H. (2016). Autoantibodies Specifically Against β1 Adrenergic Receptors and Adverse Clinical Outcome in Patients With Chronic Systolic Heart Failure in the β-Blocker Era: The Importance of Immunoglobulin G3 Subclass. J. Card. Fail..

[28] Fan Y., Chen Y., Wan Z., Zhou D., Ma A. (2017). The prognostic value of autoantibodies against β1-adrenoceptor and cardiac troponin-I for clinical outcomes in STEMI. J. Cardiovasc. Med..

[29] Brisinda D., Sorbo A.R., Venuti A., Ruggieri M.P., Manna R., Fenici P., Wallukat G., Hoebeke J., Frustaci A., Fenici R. (2012). Anti-β-adrenoceptors autoimmunity causing ‘idiopathic’ arrhythmias and cardiomyopathy. Circ. J. Off. J. Jpn. Circ. Soc..

[30] Fedorowski A., Li H., Yu X., Koelsch K.A., Harris V.M., Liles C., Murphy T.A., Quadri S.M.S., Scofield R.H., Sutton R. (2017). Antiadrenergic autoimmunity in postural tachycardia syndrome. Eur. Pacing Arrhythm. Card. Electrophysiol. J. Work. Groups Card. Pacing Arrhythm. Card. Cell. Electrophysiol. Eur. Soc. Cardiol..

[31] Becker N.P., Müller J., Göttel P., Wallukat G., Schimke I. (2017). Cardiomyopathy—An approach to the autoimmune background. Autoimmun. Rev..

[32] Schulze W., Kunze R., Wallukat G. (2005). Pathophysiological role of autoantibodies against G-protein-coupled receptors in the cardiovascular system. Exp. Clin. Cardiol..

[33] Wallukat G., Wollenberger A., Morwinski R., Pitschner H.F. (1995). Anti-beta 1-adrenoceptor autoantibodies with chronotropic activity from the serum of patients with dilated cardiomyopathy: Mapping of epitopes in the first and second extracellular loops. J. Mol. Cell. Cardiol..

[34] Nikolaev V.O., Boivin V., Störk S., Angermann C.E., Ertl G., Lohse M.J., Jahns R. (2007). A novel fluorescence method for the rapid detection of functional beta1-adrenergic receptor autoantibodies in heart failure. J. Am. Coll. Cardiol..

[35] Mobini R., Magnusson Y., Wallukat G., Viguier M., Hjalmarson A., Hoebeke J. (1999). Probing the immunological properties of the extracellular domains of the human beta(1)-adrenoceptor. J. Autoimmun..

[36] Mobini R., Fu M., Wallukat G., Magnusson Y., Hjalmarson A., Hoebeke J. (2000). A monoclonal antibody directed against an autoimmune epitope on the human beta1-adrenergic receptor recognized in idiopathic dilated cardiomyopathy. Hybridoma.

[37] Staudt A., Mobini R., Fu M., Grosse Y., Stangl V., Stangl K., Thiele A., Baumann G., Felix S.B. (2001). beta(1)-Adrenoceptor antibodies induce positive inotropic response in isolated cardiomyocytes. Eur. J. Pharmacol..

[38] Podlowski S., Luther H.P., Morwinski R., Muller J., Wallukat G. (1998). Agonistic anti-beta1-adrenergic receptor autoantibodies from cardiomyopathy patients reduce the beta1-adrenergic receptor expression in neonatal rat cardiomyocytes. Circulation.

[39] Jahns R., Boivin V., Krapf T., Wallukat G., Boege F., Lohse M.J. (2000). Modulation of beta1-adrenoceptor activity by domain-specific antibodies and heart failure-associated autoantibodies. J. Am. Coll. Cardiol..

[40] Staudt Y., Mobini R., Fu M., Felix S.B., Kuhn J.P., Staudt A. (2003). Beta1-adrenoceptor antibodies induce apoptosis in adult isolated cardiomyocytes. Eur. J. Pharmacol..

[41] Fukuda Y., Miyoshi S., Tanimoto K., Oota K., Fujikura K., Iwata M., Baba A., Hagiwara Y., Yoshikawa T., Mitamura H. (2004). Autoimmunity against the second extracellular loop of beta(1)-adrenergic receptors induces early afterdepolarization and decreases in K-channel density in rabbits. J. Am. Coll. Cardiol..

[42] Christ T., Wettwer E., Dobrev D., Adolph E., Knaut M., Wallukat G., Ravens U. (2001). Autoantibodies against the beta1 adrenoceptor from patients with dilated cardiomyopathy prolong action potential duration and enhance contractility in isolated cardiomyocytes. J. Mol. Cell. Cardiol..

[43] Medei E.H., Nascimento J.H., Pedrosa R.C., Barcellos L., Masuda M.O., Sicouri S., Elizari M.V., de Carvalho A.C. (2008). Antibodies with beta-adrenergic activity from chronic chagasic patients modulate the QT interval and M cell action potential duration. Eur. Pacing Arrhythm. Card. Electrophysiol. J. Work. Groups Card. Pacing Arrhythm. Card. Cell. Electrophysiol. Eur. Soc. Cardiol..

[44] Matsui S., Fu M.L., Hayase M., Katsuda S., Yamaguchi N., Teraoka K., Kurihara T., Takekoshi N. (1999). Active immunization of combined beta1-adrenoceptor and M2-muscarinic receptor peptides induces cardiac hypertrophy in rabbits. J. Card. Fail..

[45] Zuo L., Bao H., Tian J., Wang X., Zhang S., He Z., Yan L., Zhao R., Ma X.L., Liu H. (2011). Long-term active immunization with a synthetic peptide corresponding to the second extracellular loop of β1-adrenoceptor induces both morphological and functional cardiomyopathic changes in rats. Int. J. Cardiol..

[46] Jane-wit D., Altuntas C.Z., Johnson J.M., Yong S., Wickley P.J., Clark P., Wang Q., Popović Z.B., Penn M.S., Damron D.S. (2007). Beta 1-adrenergic receptor autoantibodies mediate dilated cardiomyopathy by agonistically inducing cardiomyocyte apoptosis. Circulation.

[47] Abdelkrim M.A., Noireaud J., Chatagnon G., Gogny M., Desfontis J.C., Mallem M.Y. (2012). Antibodies against the second extracellular loop of beta1-adrenergic receptor induce aortic endothelial dysfunction in Wistar rat. Ann. Cardiol. D’angeiol..

[48] Torre-Amione G., Orrego C.M., Khalil N., Kottner-Assad C., Leveque C., Celis R., Youker K.A., Estep J.D. (2010). Therapeutic plasma exchange a potential strategy for patients with advanced heart failure. J. Clin. Apher..

[49] Dandel M., Weng Y., Siniawski H., Potapov E., Drews T., Lehmkuhl H.B., Knosalla C., Hetzer R. (2008). Prediction of cardiac stability after weaning from left ventricular assist devices in patients with idiopathic dilated cardiomyopathy. Circulation.

[50] Iwata M., Yoshikawa T., Baba A., Anzai T., Mitamura H., Ogawa S. (2001). Autoantibodies against the second extracellular loop of beta1-adrenergic receptors predict ventricular tachycardia and sudden death in patients with idiopathic dilated cardiomyopathy. J. Am. Coll. Cardiol..

[51] Zhang L., Hu D., Li J., Wu Y., Liu X., Yang X. (2002). Autoantibodies against the myocardial beta1-adrenergic and M2-muscarinic receptors in patients with congestive heart failure. Chin. Med. J..

[52] Miao G.B., Liu J.C., Liu M.B., Wu J.L., Zhang G., Chang J., Zhang L. (2006). Autoantibody against beta1-adrenergic receptor and left ventricular remodeling changes in response to metoprolol treatment. Eur. J. Clin. Investig..

[53] Nagatomo Y., Yoshikawa T., Kohno T., Yoshizawa A., Baba A., Anzai T., Meguro T., Satoh T., Ogawa S. (2009). A pilot study on the role of autoantibody targeting the beta1-adrenergic receptor in the response to beta-blocker therapy for congestive heart failure. J. Card. Fail..

[54] Nagatomo Y., Yoshikawa T., Okamoto H., Kitabatake A., Hori M. (2015). Presence of autoantibody directed against β1-adrenergic receptors is associated with amelioration of cardiac function in response to carvedilol: Japanese Chronic Heart Failure (J-CHF) Study. J. Card. Fail..

[55] Pei J., Li N., Chen J., Li X., Zhang Y., Wang Z., Zhang P., Cao K., Pu J. (2012). The predictive values of beta1-adrenergic and M2 muscarinic receptor autoantibodies for sudden cardiac death in patients with chronic heart failure. Eur. J. Heart Fail..

[56] Markousis-Mavrogenis G., Minich W.B., Al-Mubarak A.A., Anker S.D., Cleland J.G.F., Dickstein K., Lang C.C., Ng L.L., Samani N.J., Zannad F. (2023). Clinical and prognostic associations of autoantibodies recognizing adrenergic/muscarinic receptors in patients with heart failure. Cardiovasc. Res..

[57] Nagatomo Y., McNamara D.M., Alexis J.D., Cooper L.T., Dec G.W., Pauly D.F., Sheppard R., Starling R.C., Tang W.H. (2017). Myocardial Recovery in Patients With Systolic Heart Failure and Autoantibodies Against β(1)-Adrenergic Receptors. J. Am. Coll. Cardiol..

[58] (1999). The Cardiac Insufficiency Bisoprolol Study II (CIBIS-II): A randomised trial. Lancet.

[59] (1999). Effect of metoprolol CR/XL in chronic heart failure: Metoprolol CR/XL Randomised Intervention Trial in Congestive Heart Failure (MERIT-HF). Lancet.

[60] Packer M., Fowler M.B., Roecker E.B., Coats A.J., Katus H.A., Krum H., Mohacsi P., Rouleau J.L., Tendera M., Staiger C. (2002). Effect of carvedilol on the morbidity of patients with severe chronic heart failure: Results of the carvedilol prospective randomized cumulative survival (COPERNICUS) study. Circulation.

[61] McDonagh T.A., Metra M., Adamo M., Gardner R.S., Baumbach A., Böhm M., Burri H., Butler J., Čelutkienė J., Chioncel O. (2021). 2021 ESC Guidelines for the diagnosis and treatment of acute and chronic heart failure. Eur. Heart J..

[62] Heidenreich P.A., Bozkurt B., Aguilar D., Allen L.A., Byun J.J., Colvin M.M., Deswal A., Drazner M.H., Dunlay S.M., Evers L.R. (2022). 2022 AHA/ACC/HFSA Guideline for the Management of Heart Failure: A Report of the American College of Cardiology/American Heart Association Joint Committee on Clinical Practice Guidelines. Circulation.

[63] Du Y., Yan L., Wang J., Zhan W., Song K., Han X., Li X., Cao J., Liu H. (2012). β1-Adrenoceptor autoantibodies from DCM patients enhance the proliferation of T lymphocytes through the β1-AR/cAMP/PKA and p38 MAPK pathways. PLoS ONE.

[64] Dörffel W.V., Felix S.B., Wallukat G., Brehme S., Bestvater K., Hofmann T., Kleber F.X., Baumann G., Reinke P. (1997). Short-term hemodynamic effects of immunoadsorption in dilated cardiomyopathy. Circulation.

[65] Felix S.B., Staudt A., Dörffel W.V., Stangl V., Merkel K., Pohl M., Döcke W.D., Morgera S., Neumayer H.H., Wernecke K.D. (2000). Hemodynamic effects of immunoadsorption and subsequent immunoglobulin substitution in dilated cardiomyopathy: Three-month results from a randomized study. J. Am. Coll. Cardiol..

[66] Müller J., Wallukat G., Dandel M., Bieda H., Brandes K., Spiegelsberger S., Nissen E., Kunze R., Hetzer R. (2000). Immunoglobulin adsorption in patients with idiopathic dilated cardiomyopathy. Circulation.

[67] Knebel F., Böhm M., Staudt A., Borges A.C., Tepper M., Jochmann N., Wernicke K.D., Felix S., Baumann G. (2004). Reduction of morbidity by immunoadsorption therapy in patients with dilated cardiomyopathy. Int. J. Cardiol..

[68] Dörffel W.V., Wallukat G., Dörffel Y., Felix S.B., Baumann G. (2004). Immunoadsorption in idiopathic dilated cardiomyopathy, a 3-year follow-up. Int. J. Cardiol..

[69] Camino M., Morales M.D. (2019). Beta(1)-adrenergic receptor antibodies in children with dilated cardiomyopathy. Front. Biosci..

[70] Dandel M., Wallukat G., Englert A., Lehmkuhl H.B., Knosalla C., Hetzer R. (2012). Long-term benefits of immunoadsorption in β(1)-adrenoceptor autoantibody-positive transplant candidates with dilated cardiomyopathy. Eur. J. Heart Fail..

[71] Wallukat G., Müller J., Hetzer R. (2002). Specific removal of beta1-adrenergic autoantibodies from patients with idiopathic dilated cardiomyopathy. New Engl. J. Med..

[72] Schimke I., Muller J., Dandel M., Gremmels H.D., Bayer W., Wallukat B., Wallukat G., Hetzer R. (2005). Reduced oxidative stress in parallel to improved cardiac performance one year after selective removal of anti-beta 1-adrenoreceptor autoantibodies in patients with idiopathic dilated cardiomyopathy: Data of a preliminary study. J. Clin. Apher..

[73] Stavrakis S., Kem D.C., Patterson E., Lozano P., Huang S., Szabo B., Cunningham M.W., Lazzara R., Yu X. (2011). Opposing cardiac effects of autoantibody activation of β-adrenergic and M2 muscarinic receptors in cardiac-related diseases. Int. J. Cardiol..

[74] Nagatomo Y., Baba A., Ito H., Naito K., Yoshizawa A., Kurita Y., Nakamura I., Monkawa T., Matsubara T., Wakabayashi Y. (2011). Specific immunoadsorption therapy using a tryptophan column in patients with refractory heart failure due to dilated cardiomyopathy. J. Clin. Apher..

[75] Yoshikawa T., Baba A., Akaishi M., Wakabayashi Y., Monkawa T., Kitakaze M., Izumi T., Tomoike H. (2016). Immunoadsorption therapy for dilated cardiomyopathy using tryptophan column-A prospective, multicenter, randomized, within-patient and parallel-group comparative study to evaluate efficacy and safety. J. Clin. Apher..

[76] Cooper L.T., Belohlavek M., Korinek J., Yoshifuku S., Sengupta P.P., Burgstaler E.A., Winters J.L. (2007). A pilot study to assess the use of protein a immunoadsorption for chronic dilated cardiomyopathy. J. Clin. Apher..

[77] Mobini R., Staudt A., Felix S.B., Baumann G., Wallukat G., Deinum J., Svensson H., Hjalmarson A., Fu M. (2003). Hemodynamic improvement and removal of autoantibodies against beta1-adrenergic receptor by immunoadsorption therapy in dilated cardiomyopathy. J. Autoimmun..

[78] Staudt A., Bohm M., Knebel F., Grosse Y., Bischoff C., Hummel A., Dahm J.B., Borges A., Jochmann N., Wernecke K.D. (2002). Potential role of autoantibodies belonging to the immunoglobulin G-3 subclass in cardiac dysfunction among patients with dilated cardiomyopathy. Circulation.

[79] Staudt A., Dörr M., Staudt Y., Böhm M., Probst M., Empen K., Plötz S., Maschke H.E., Hummel A., Baumann G. (2005). Role of immunoglobulin G3 subclass in dilated cardiomyopathy: Results from protein A immunoadsorption. Am. Heart J..

[80] Baba A., Akaishi M., Shimada M., Monkawa T., Wakabayashi Y., Takahashi M., Nagatomo Y., Yoshikawa T. (2010). Complete elimination of cardiodepressant IgG3 autoantibodies by immunoadsorption in patients with severe heart failure. Circ. J. Off. J. Jpn. Circ. Soc..

[81] Jahns R., Boivin V., Siegmund C., Boege F., Lohse M.J., Inselmann G. (1999). Activating beta-1-adrenoceptor antibodies are not associated with cardiomyopathies secondary to valvular or hypertensive heart disease. J. Am. Coll. Cardiol..

[82] Bornholz B., Weidtkamp-Peters S., Schmitmeier S., Seidel C.A., Herda L.R., Felix S.B., Lemoine H., Hescheler J., Nguemo F., Schafer C. (2013). Impact of human autoantibodies on beta1-adrenergic receptor conformation, activity, and internalization. Cardiovasc. Res..

[83] Xu B.Y., Pirskanen R., Lefvert A.K. (1998). Antibodies against beta1 and beta2 adrenergic receptors in myasthenia gravis. J. Neuroimmunol..

[84] Fujii H., Sato W., Kimura Y., Matsuda H., Ota M., Maikusa N., Suzuki F., Amano K., Shin I., Yamamura T. (2020). Altered Structural Brain Networks Related to Adrenergic/Muscarinic Receptor Autoantibodies in Chronic Fatigue Syndrome. J. Neuroimaging Off. J. Am. Soc. Neuroimaging.

[85] Segovia M., Ganzinelli S., Reina S., Borda E., Sterin-Borda L. (2012). Role of anti-β1 adrenergic antibodies from patients with periodontitis in cardiac dysfunction. J. Oral Pathol. Med. Off. Publ. Int. Assoc. Oral Pathol. Am. Acad. Oral Pathol..

[86] Reina S., Ganzinelli S., Sterin-Borda L., Borda E. (2012). Pro-apoptotic effect of anti-β1-adrenergic receptor antibodies in periodontitis patients. Int. Immunopharmacol..

[87] Kaneko M., Swanson M.C., Gleich G.J., Kita H. (1995). Allergen-specific IgG1 and IgG3 through Fc gamma RII induce eosinophil degranulation. J. Clin. Investig..

[88] Mijares A., Lebesgue D., Wallukat G., Hoebeke J. (2000). From agonist to antagonist: Fab fragments of an agonist-like monoclonal anti-beta(2)-adrenoceptor antibody behave as antagonists. Mol. Pharmacol..

[89] Staudt A., Eichler P., Trimpert C., Felix S.B., Greinacher A. (2007). Fc(gamma) receptors IIa on cardiomyocytes and their potential functional relevance in dilated cardiomyopathy. J. Am. Coll. Cardiol..

[90] Staudt A., Herda L.R., Trimpert C., Lubenow L., Landsberger M., Dörr M., Hummel A., Eckerle L.G., Beug D., Müller C. (2010). Fcgamma-receptor IIa polymorphism and the role of immunoadsorption in cardiac dysfunction in patients with dilated cardiomyopathy. Clin. Pharmacol. Ther..

[91] Ernst D., Westerbergh J., Sogkas G., Jablonka A., Ahrenstorf G., Schmidt R.E., Heidecke H., Wallentin L., Riemekasten G., Witte T. (2019). Lowered anti-beta1 adrenergic receptor antibody concentrations may have prognostic significance in acute coronary syndrome. Sci. Rep..

[92] Chiale P.A., Garro H.A., Schmidberg J., Sánchez R.A., Acunzo R.S., Lago M., Levy G., Levin M. (2006). Inappropriate sinus tachycardia may be related to an immunologic disorder involving cardiac beta andrenergic receptors. Heart Rhythm..

[93] Hu B., Sun Y., Li S., Sun J., Liu T., Wu Z., Feng L.I. (2016). Association of β1-Adrenergic, M2-Muscarinic Receptor Autoantibody with Occurrence and Development of Nonvalvular Atrial Fibrillation. Pacing Clin. Electrophysiol..

[94] Yalcin M.U., Gurses K.M., Kocyigit D., Kesikli S.A., Ates A.H., Evranos B., Yorgun H., Sahiner M.L., Kaya E.B., Oto M.A. (2015). Elevated M2-muscarinic and β1-adrenergic receptor autoantibody levels are associated with paroxysmal atrial fibrillation. Clin. Res. Cardiol. Off. J. Ger. Card. Soc..

[95] Stavrakis S., Yu X., Patterson E., Huang S., Hamlett S.R., Chalmers L., Pappy R., Cunningham M.W., Morshed S.A., Davies T.F. (2009). Activating autoantibodies to the beta-1 adrenergic and m2 muscarinic receptors facilitate atrial fibrillation in patients with Graves’ hyperthyroidism. J. Am. Coll. Cardiol..

[96] Galloway A., Li H., Vanderlinde-Wood M., Khan M., Benbrook A., Liles C., Zillner C., Rao V., Cunningham M.W., Yu X. (2015). Activating autoantibodies to the β1/2-adrenergic and M2 muscarinic receptors associate with atrial tachyarrhythmias in patients with hyperthyroidism. Endocrine.

[97] Sun H., Song J., Li K., Li Y., Shang L., Zhou Q., Lu Y., Zong Y., He X., Kari M. (2023). Increased β1-adrenergic receptor antibody confers a vulnerable substrate for atrial fibrillation via mediating Ca2+ mishandling and atrial fibrosis in active immunization rabbit models. Clin. Sci..

[98] Li H., Murphy T., Zhang L., Huang B., Veitla V., Scherlag B.J., Kem D.C., Yu X. (2016). β1-Adrenergic and M2 Muscarinic Autoantibodies and Thyroid Hormone Facilitate Induction of Atrial Fibrillation in Male Rabbits. Endocrinology.

[99] Li H., Zhang G., Zhou L., Nuss Z., Beel M., Hines B., Murphy T., Liles J., Zhang L., Kem D.C. (2019). Adrenergic Autoantibody-Induced Postural Tachycardia Syndrome in Rabbits. J. Am. Heart Assoc..

[100] Gunning W.T., Kvale H., Kramer P.M., Karabin B.L., Grubb B.P. (2019). Postural Orthostatic Tachycardia Syndrome Is Associated With Elevated G-Protein Coupled Receptor Autoantibodies. J. Am. Heart Assoc..

[101] Ke F., Kuang W., Hu X., Li C., Ma W., Shi D., Li X., Wu Z., Zhou Y., Liao Y. (2023). A novel vaccine targeting β1-adrenergic receptor. Hypertens. Res. Off. J. Jpn. Soc. Hypertens..

[102] Wallukat G., Haberland A., Berg S., Schulz A., Freyse E.J., Dahmen C., Kage A., Dandel M., Vetter R., Salzsieder E. (2012). The first aptamer-apheresis column specifically for clearing blood of beta1-receptor autoantibodies. Circ. J. Off. J. Jpn. Circ. Soc..

[103] Düngen H.D., Dordevic A., Felix S.B., Pieske B., Voors A.A., McMurray J.J.V., Butler J. (2020). β(1)-Adrenoreceptor Autoantibodies in Heart Failure: Physiology and Therapeutic Implications. Circulation. Heart Fail..

[104] Konishi M., Ishida J., Springer J., von Haehling S., Akashi Y.J., Shimokawa H., Anker S.D. (2016). Heart failure epidemiology and novel treatments in Japan: Facts and numbers. ESC Heart Fail..

[105] Plosker G.L., Figgitt D.P. (2003). Rituximab: A review of its use in non-Hodgkin’s lymphoma and chronic lymphocytic leukaemia. Drugs.

[106] Tschöpe C., Van Linthout S., Spillmann F., Posch M.G., Reinke P., Volk H.D., Elsanhoury A., Kühl U. (2019). Targeting CD20+ B-lymphocytes in inflammatory dilated cardiomyopathy with rituximab improves clinical course: A case series. Eur. Heart J. Case Rep..

